# Identification of RSPO2 Fusion Mutations and Target Therapy Using a Porcupine Inhibitor

**DOI:** 10.1038/s41598-018-32652-3

**Published:** 2018-09-24

**Authors:** Chong Li, Jing Cao, Ning Zhang, Meiqing Tu, Fengwei Xu, Shuang Wei, Xiaojing Chen, Yuhong Xu

**Affiliations:** Pharmacy School, Shanghai Jiaotong University, Shanghai, 200240 China

## Abstract

Cancers are driven by a variety of somatic gene mutations and identifying these mutations enables the development of novel target drugs. We have sought to identify abnormalities in Wnt pathway-related genes that are sensitive to Wnt inhibitor treatment. We examined Patient Derived Xenograft (PDX) RNA samples and found new R-Spondin 2 (RSPO2) transcript fusions with the EMC2, PVT1 or HNF4G genes. These fusion events were identified in about 1.4% of the digestive system cancer samples. We then examined the oncogenic effects of the RSPO2-EMC2 fusion gene and confirmed that it can drive oncogenesis, sustain tumor growth and promote metastasis. Finally, we used a Wnt pathway Porcupine inhibitor CGX1321 to treat PDX mouse models containing RSPO2 fusion genes. All the RSPO2 fusion tumors responded to the treatment and stopped progression. Our data show that Wnt pathway inhibition could provide an effective treatment for cancers containing RSPO2 fusion. The RSPO2 fusion will serve as a good biomarker for screening patients to support clinical treatment of digestive system cancers using Wnt pathway inhibitors.

## Introduction

The Wnt pathway is a key developmental pathway involved in multiple types of cancer^1,2^, including liver^3^, colon^4,5^, and lung cancers^6,7^. There are two Wnt pathways: the canonical Wnt/β-catenin signaling and noncanonical signaling pathways^8,9^. In the classical canonical Wnt signaling pathway, the transcriptional regulator β-catenin remains under control of the destruction complex consisting of APC, AXIN2, and GSK3β^10^. Upon activation, β-catenin translocates to the nucleus and docks at the TCF/LEF-binding sites to activate downstream gene expression^11,12^. Such activation requires multiple protein complexation, which includes the Wnt ligand as well as^13,14^, Frizzled^15^ and RNF43^16^. In 2004, Kazankaya *et al*. also showed that RSPOs act as extracellular factors that promote Wnt/β-catenin signaling in HEK293T cells^17^. Recently, it was reported that RSPOs could bind to LGR4, LGR5 and LGR6 simultaneous and remarkably enhanced the canonical Wnt Signaling^18,19^. The ligand-receptor interaction of RSPOs and LGRs exerted their potentiating effects through direct binding with the extracellular regions of ZNRF3 or RNF43^20^ and interfered with the ubiquitination of the Frizzled receptors and their degradation^21,22^. Considering the important roles of RSPOs in Canonical Wnt pathway activities, several studies have examined RSPO gene mutations and investigated their involvement in cancer development. Two previous studies found RSPO2 transcripts fusions and RSPO2 over-expression in colon cancer patients^23,24^. Seshagiri *et al*. reported RSPO fusion mutation with EIF3F and PTPRK genes, but these RSPO fusions were mutually exclusive with mutations in β-catenin and APC. In RSPO fusion positive colon cancers, Wnt pathway target-genes are up-regulated, similar to tumors carrying APC mutation^23^. Studies of the PTPRK-RSPO3 fusion has prompted the developments of RSPO3 antibodies and other Wnt pathway inhibitor, using RSPO3 fusion as a biomarker in the treatment^25,26^.

Here, we analyzed various types of RSPO2 fusions in PDX tumors and confirmed their driving effects in tumor progression. RSPO2 fusions exhibit oncogene function in studies *in vivo* and *in vitro*. Treatment of RSPO2 fusion tumors *in vivo* with a Wnt inhibitor resulted in growth inhibition. These results indicate that RSPO2 fusions may serve as a good biomarker for patient selection in gastric and colon cancers that are dependent on the canonical Wnt pathway^27^.

## Results

### Characterization of RSPO2 fusions in PDX tumors

**Figure 1:**
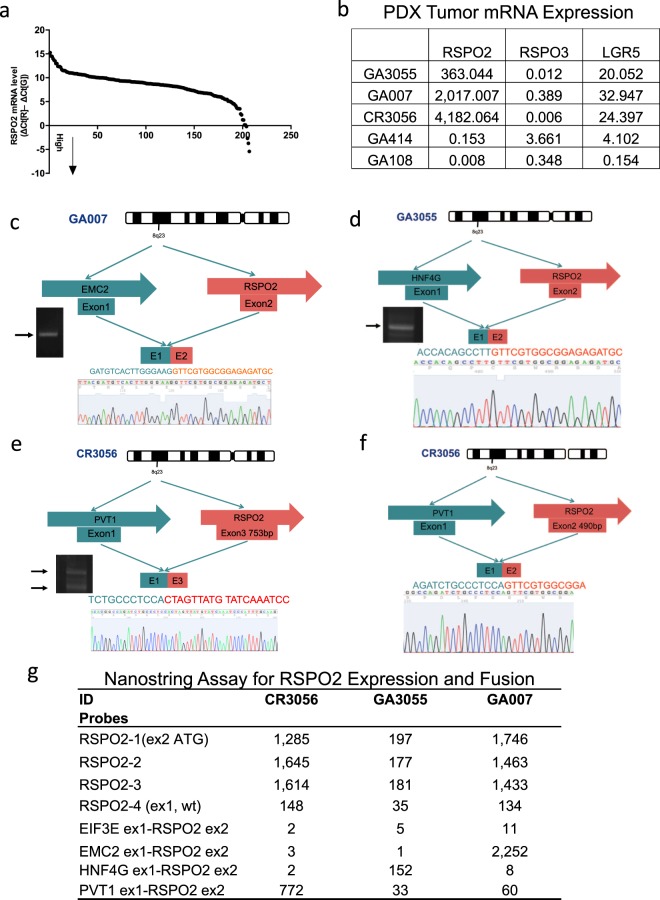
RSPO2 fusion genes found in three human tumor tissues. (**a**) RSPO2 mRNA levels from PDX tumor samples tested by q-PCR assay. (**b**) RSPO2, RSPO3 and LGR5 mRNA expression in GA007, GA3055, CR3056, GA108, and GA414 samples. (**c**) EMC2 exon 2-RSPO2 exon 1 fusion in GA007. (**d**) HNF4 exon 1-RSPO2 exon 2 fusion in GA3055. (**e**,**f**) PVT1 exon 1-RSPO2 exon 1 fusion and PVT1 exon 1-RSPO2 exon 3 fusion in CR3056. (**g**) Nanostring assay of various RSPO2 fusion genes and expression in GA007, GA3055 and CR3056 samples.

We performed RSPO2 expression screening using RNA samples in a PDX library containing gastric, colon, liver, pancreatic and lung cancer tumor samples, with a total of 207 PDX samples. RSPO2 mRNA levels were quantified by q-PCR and plotted in Fig. 1a. We listed the detailed RSPO2 copy data of five of the samples in Fig. 1b, along with q-PCR data for RSPO3 and LGR5. Three samples had elevated RSPO2 mRNA levels, including GA007 (Gastric cancer 007), GA3055 (Gastric cancer 3055), and CR3056 (Colorectal cancer 3056). In contrast, GA108 and GA414 had relatively low levels of RSPO2 expression.

To identify the cause of RSPO2 over expression, we used RACE (rapid amplification of cDNA ends) PCR to trace the 5′ upstream sequence of RSPO2 by designing a Gen-Specific Primer (GSP) targeting the exon 3 region of RSPO2. The RACE PCR products were analyzed using agarose gel electrophoresis. They were subsequently cloned using the Infusion HD cloning kit and sequenced. The results revealed several different somatic fusions of the RSPO2 gene (Fig. 1c–f).

In the GA007 gastric cancer sample, we identified a fusion between the EMC2 gene exon 1 (NM_014673.4) and the RSPO2 exon 2 (NM_178565.4). This gene fusion lacked the EMC2 exon 2 and RSPO2 exon 1 (Fig. 1c). The fusion point within RSPO2 exon 2 (at 490 bp) is the same as that in the EIF3E exon1-RSPO2 exon2 fusion discovered by Seshagiri and coworkers^23^. Because the ATG of RSPO2 is still within Exon2, the fusion partner acts mainly as strong promoter. The actual protein produced is still native RSPO2.

The GA3055 tumor contained an HNF4G-RSPO2 fusion between RSPO2 exon 2 and HNF4G exon 3 (NM_001330561.1) (Fig. 1d). The fusion point is at exon 2 (490 bp) of RSPO2 and exon 3 of HNF4G (at 447 bp). The ATG of RSPO2 is unaltered. The actual protein produced is still native RSPO2 as the same as the EMC2-RSPO2 fusion.

The CR3056 tumor contained multiple fusion transcripts copies, as indicated by the presence of two PCR bands in Fig. 1e. We identified a fusion event between PVT1 gene exon 1 (at 202 bp) (NR_003367.3) and RSPO2 exon 2 (490 bp) as well as a fusion event between PVT1 exon 1 and RSPO2 exon 3 (at 753 bp) (Fig. 1e,f). The PVT1 exon 1-RSPO2 exon 3 fusion does not produce functional protein due to frameshift and premature stop codon in RSPO2 exon 3. The PVT1-RSPO2 exon 2 fusion does produce a functional protein and has higher levels of RSPO2 expression.

We confirmed these results using a Nanostring assay to quantify the various gene sequences, including all the fusion found in this study and the EIF3E-RSPO2 fusion reported previously. Nanostring directly measures gene copies without RNA reverse transcription and PCR. The presence of fusion gene indeed resulted in highly elevated RSPO2 gene copies as shown in Fig. 1h.

### EMC2-RSPO2 fusion gene drives tumor progression via Wnt pathway signaling

**Figure 2:**
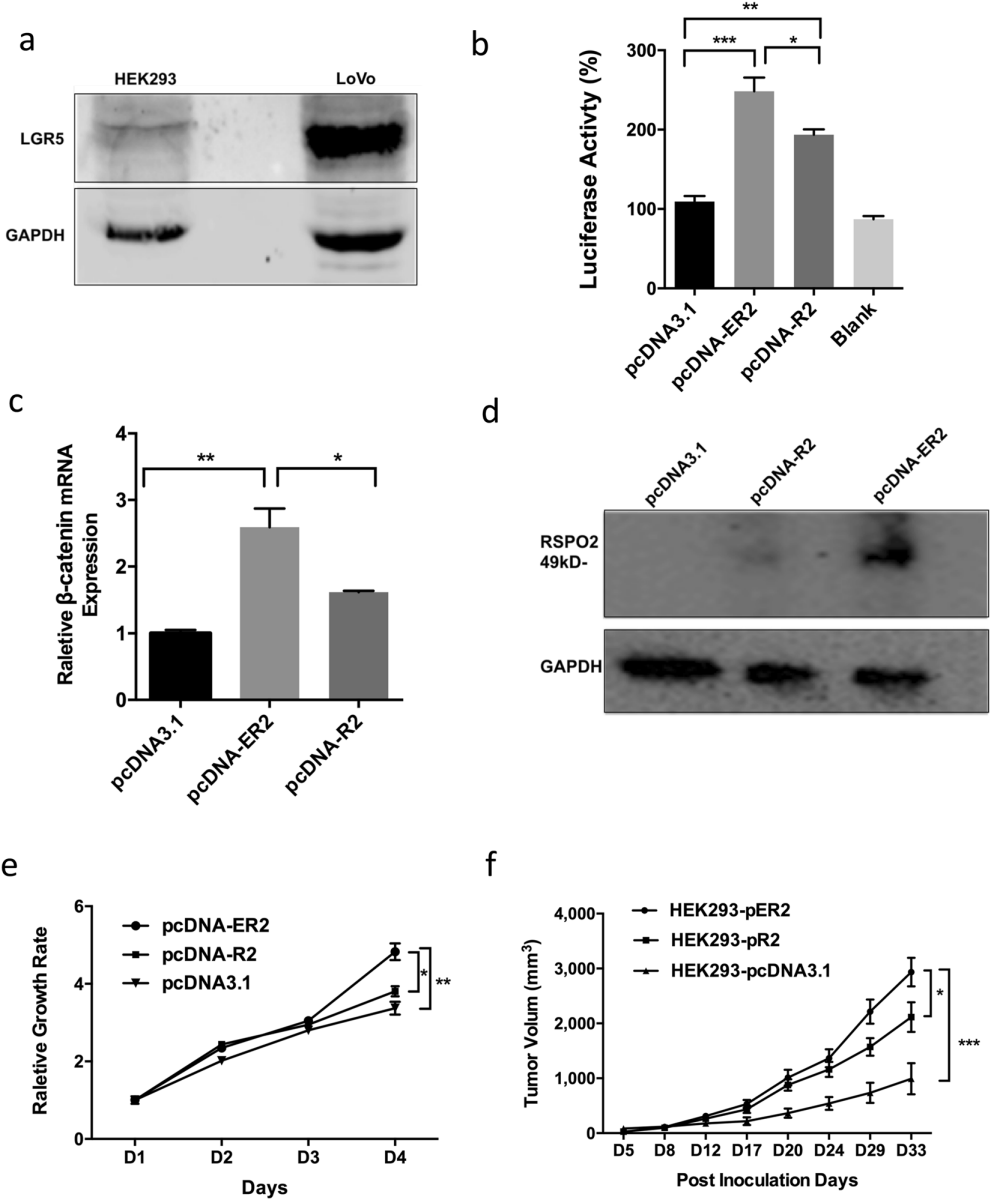
The effect of EMC2-RSPO2 fusion gene expression in HEK293 cells. (**a**) Western Blot assay of LGR5 protein in HEK293 and LoVo cells. (**b**) WNT/β-catenin pathway activities in HEK293- TCF cells transfected with pcDNA-R2, pcDNA-ER2 and pcDNA3.1 plasmid. (**c**) β-catenin expression by q-PCR detection in HEK293 after transfection of pcDNA-R2, pcDNA-ER2 and pcDNA3.1. (**d**) RSPO2 protein expression in HEK293 after transfection of pcDNA-R2, pcDNA-ER2 and pcDNA3. (**e**) Cell proliferation curves of HEK293 stably transfected by plasmid pcDNA-R2, pcDNA-ER2 and pcDNA3. (**f**) Tumor growth curves by the HEK293-pER2, HEK293-pR2, HEK293-pcDNA3.1 induced by implanting 5×10^6^ cells in nude mice. Mice number N = 10. *p < 0.05, **p < 0.01, ***p < 0.001.

HEK293T cells and LoVo cells have constitutive expression of the RSPO receptor LGR5 (Fig. 2a). To investigate the RSPO2 fusion gene function, the RSPO2-EMC2 fusion gene was cloned into a pcDNA3.1 expression plasmid, denoted as pcDNA-ER2 (pcDNA-EMC2-RSPO2). pcDNA-R2 (pcDNA-RSPO2) and pcDNA3.1 plasmids were cloned for comparison. The plasmids were transfected into HEK293-TCF cells where luciferase expression is regulated by the TCF/LEF promoter^28^, resulting in significantly higher Wnt pathway activity in cells transfected with pcDNA-ER2 and pcDNA-R2 (Fig. 2b). β-catenin mRNA expression level increased in pcDNA-ER2 transfected cells as well (Fig. 2c). Because the starting codon of RSPO2 is located in exon 2 and is retained in the EMC2-RSPO2 fusion gene, the RSPO2 protein sequence was not affected. But RSPO2 transcription may be regulated by 5′UTR in exon 1 of EMC2, resulting in high RSPO2 protein expression (Fig. 2d). The pcDNA-R2 or pcDNA-ER2 plasmids were stably transfected into HEK293 cells for cell proliferation studies. Proliferation of pcDNA-ER2 transfected cells was faster than proliferation of cells transfected with pcDNA-R2 or pcDNA3.1 (Fig. 2e). These cells were also injected into nude mice (5 × 10^6^ cells per mouse). The mice receiving EMC2-RSPO2 transfected cells showed an increase in tumor growth rate (Fig. 2f).

### Excessive RSPO2 promoted LGR5 +cell proliferation and migration

**Figure 3:**
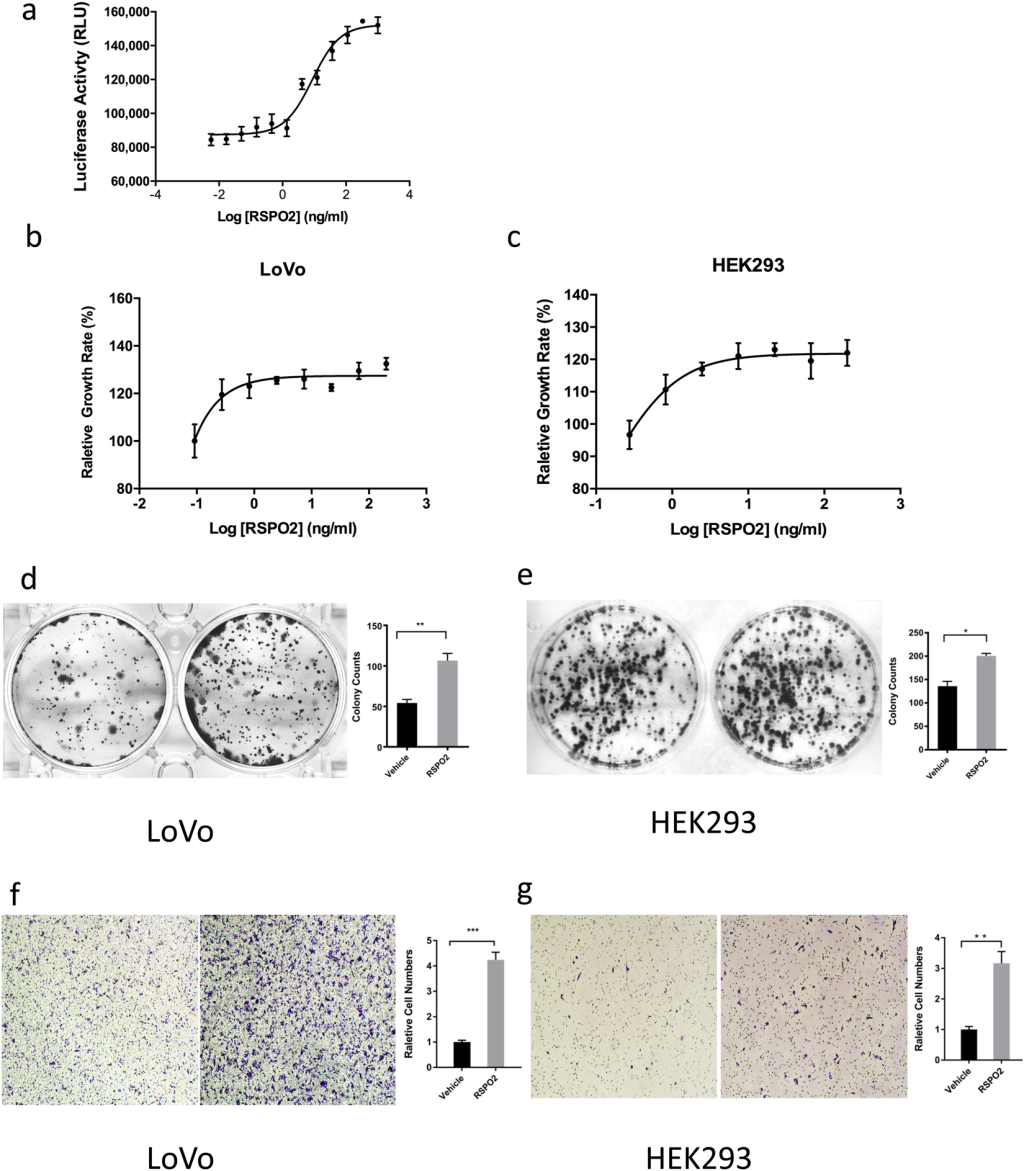
The elevation of LGR5 + cell proliferation and migration by RSPO2. (**a**) RSPO2 enhances the WNT pathway activity in dosing dependent manner, the EC_50_ = 8.65 ng/mL. (**b**,**c**) Cell proliferation curves of HEK293 and LoVo cells activated by culture medium containing 10 ng/mL RSPO proteins. (**d**,**e**) HEK293 and LoVo Foci formation assay, Left: control group, Right: experimental group with 10 ng/mL RSPO2 added in cell culture medium. (**f**,**g**) HEK293 and LoVo transwell migration assay, Left: vehicle group, Right: experimental group containing 10 ng/mL RSPO2 in cell culture medium. *p < 0.05, **p < 0.01, ***p < 0.001.

Because our fusion genes did not alter the RSPO2 expression cassette, gene modifications only affected RSPO2 expression and secretion in tumor tissues. To investigate the consequences of higher levels of RSPO2, we co-cultured RSPO2 protein with HEK293-TCF cells, which stimulated Wnt pathway signaling in a dose-depended manner with an EC_50_ of 8.65 ng/mL (Fig. 3a). LoVo and HEK293 cell proliferation curves also showed a dose-dependent response (Fig. 3b,c). We also examined the effects of RSPO2 in cell colonies formation. In a foci formation assay, the presence of RSPO2 protein resulted in significantly higher numbers of cell colonies (Fig. 2d,e). In addition, RSPO2 promoted cell migration in both cell models (Fig. 2f,g). These data suggest that RSPO2 increases LGR5 + phenotype and behavior through the Wnt pathway.

### The inhibition of RSPO2 fusion tumor growth by PORCN inhibitor

**Figure 4:**
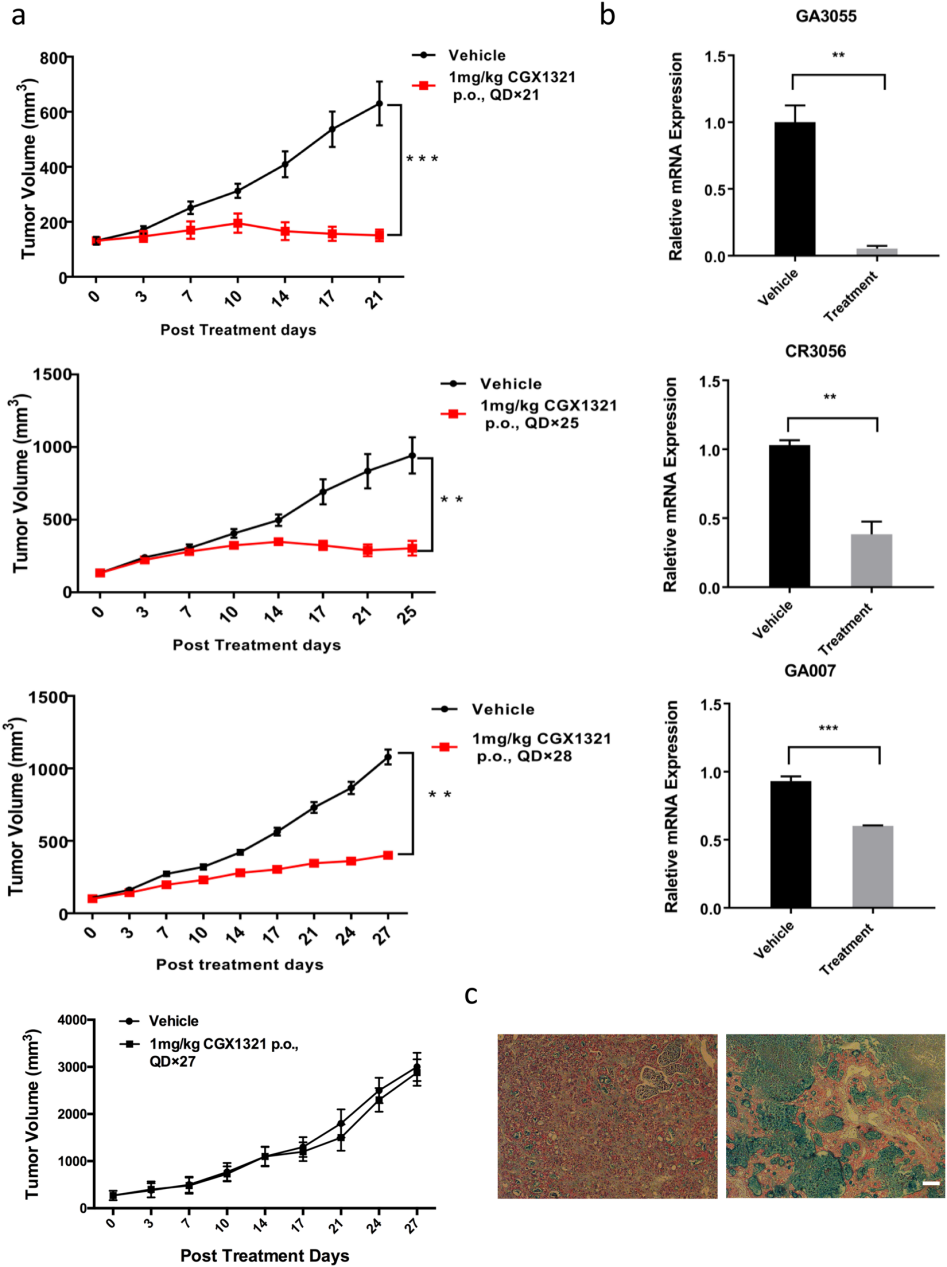
RSPO2 fusion PDX tumor growth inhibition by CGX1321. (**a**) GA3055, CR3056, GA007 and GA108 PDX xenograft mouse models were treated with 1 ml/kg vehicle formulation or 1 mg/kg CGX1321, p.o., QD for 21, 25 and 28 days respectively (N = 6). (**b**) Axin2 mRNA levels detected by quantitative RT-PCR 7 hours after final dosing (N = 4), **p < 0.01, ***p < 0.001. (**c**) Alcian blue staining of the vehicle and treated GA007 tumor samples. GA007 PDX xenograft mouse models were treated with 1 ml/kg vehicle formulation or 1 mg/kg CGX1321 for 27 days. left: vehicle group; right: treatment group. Scale bar is 50 μm.

For tumors containing RSPO2 fusion genes, we examined the therapeutic efficacy of Wnt pathway inhibitor CGX1321. CGX1321 is a PORCN (Porcupine O-Acyltransferase) inhibitor which blocks Wnt secretions. We first established a PDX nude mice model, and orally administrated CGX1321 daily to tumor-bearing mice. PDX mice models derived from GA007, CR3056, and GA3055 tumors were all responsive to treatment with CGX1321, while GA108 was not (Fig. 4a). In addition, Axin2 mRNA levels were used as a marker for Wnt pathway activity. mRNA levels of Axin in all three PDX tumors decreased after CGX1321 treatment (Fig. 4b). Alcian blue staining of the GA007 tumor showed extensive mucinous differentiation after CGX1321 treatment (Fig. 4c).

## Discussion

Abnormalities in the Wnt pathway are well known in the growth and development of various tumors^28^. There have been many investigations and explorations of targeting the Wnt pathway as a major strategy to pursue its therapeutic applications^29^. There are currently many Wnt inhibitors being developed^26,30–32^, including antibodies against LRP6 and FZD (co-receptors of Wnt)^32^ and compounds, such as LGK794 and IWP2, which are inhibitors of PORCN^33,34^. Most anti-tumor strategies targeting the Wnt pathway are focused on gene mutations that contribute to the formation or development of tumors. In a LGK794 pre-clinical study, scientists discovered that mutations of RNF43 in pancreatic ductal cancer confer Wnt dependency and sensitivity to LGK794 treatment^35^.

Here, we propose that RSPO2 is a positive regulator of Wnt pathway. RSPO2 fusion tumors are sensitive to PORCN inhibitor CGX1321 treatment through Wnt pathway inhibition. RSPO2 is a LGR5 ligand play an important role in the Wnt pathway^36^. RSPO2 is often mutated in cancers and gene fusions regulated by new promoters often lead to RSPO2 overexpression^23,24,26^. Recognizing the role of RSPO2 fusion as drivers of tumorigenesis, we show that CGX1321 treatment blocks Wnt signaling and stops LGR5 +cell growth and proliferation.

There are currently six types of RSPO2 fusions identified. EIF3F Exon 1-RSPO2 exon 2 and EIF3F exon 1-RSPO2 exon 3 fusions were identified by Seshagiri *et al*. in 2014. Our studies found four new RSPO2 fusions: EMC2 exon 1-RSPO2 exon 2, PVT1 exon 1-RSPO2 exon 2, PVT1 exon 1-RSPO2 exon 3, and HNF4G exon 1-RSPO2 exon 3. However, not all of these RSPO2 fusions are in an ORF (open reading frame). In CR3056 tumor samples, the PVT1-RSPO2 exon 3 fusion contained the fusion point starting at the exon 3 region of RSPO2, which results in frameshift and premature stop codon. Thus, PVT1 exon 1-RSPO2 exon 3 fusion is not functional. However, we identified a second in-frame PVT1-RSPO2 exon 2 fusion in the same sample, resulting high levels of RSPO2 expression. This is the first reported case of two different RSPO rearrangements co-existed in one tumor sample and its significance remains to be explored.

Using these gene fusions, we also demonstrated that elevated RSPO2 expression could drive tumorigenesis. Previous studies based on next generation sequencing suggest that RSPO fusions are mutually exclusive with APC mutations^23^. Indeed, the APC gene in GA007 was wildtype, and there was a D1822V mutation in CR3056 and GA3055. The D1822V mutation is regarded to have no correlation with cancer risk^37,38^. Therefore, our finding supports the mutual exclusivity between RSPO2 fusion and tumorigenic APC mutations. Earlier studies have also suggested that LGR5 +cells had the propensity to become cancer stem cells with APC abnormalities^39^. Here we reported that RSPO2 fusion with EMC2 resulted in LGR5 +cell proliferation and migration (Fig. 3). These LGR5 +cells play critical roles as CSCs in tumor development. Tumors containing RSPO2 fusion as described can be treated using an PORCN inhibitor CGX1321. The drug treatment could block Wnt signaling and stop tumor progression completely (Fig. 4).

In summary, we have identified novel RSPO2 fusions and demonstrated that CGX1321 has remarkable efficacy in reducing tumor growth in PDX mouse models containing RSPO2 fusion. In screening 207 PDX models with tumor samples of Asian cancer patients, we have found that about 1.4% of tumor samples contain RSPO2 fusion. This number is meaningful in pre-screening patients in clinic settings. RSPO fusions could serve as a predictive biomarker to identify cancer patients who may benefit from the treatments of CGX1321.

## Method and Materials

### Plasmid

The expression plasmid pcDNA3.1 with CMV promoter from GenScript (Nanjing) was used to construct EMC2 (NM_014673.4)-RSPO2 (NM_178565.4) fusion gene, containing EMC2-RSPO2 fusion or RSPO2. The full-length human RSPO2 and RSPO2-EMC2 cDNA were synthesized by GenScript. The pcDNA plasmid were cloned by RSPO2/ RSPO2-EMC2 denoted as pcDNA-R2/ pcDNA-ER2.

### Analysis of PDX mRNA Samples

Patient derived xenograft (PDX) frozen samples were prepared and provided by Shanghai LIDE Biotech Co., Ltd and CrownBio Co., Ltd. They obtained these samples after approval by the ethic committee, and with consents from patients. All the procedures were performed according to their Human Samples Procedure Guideline and Policies. RNeasy mini kit (Qiagen) was used for mRNA isolation from PDX frozen tissue samples or HEK293/LoVo cells. The iScript^TM^ cDNA Synthesis Kit (BioRad) was used for first-strand cDNA synthesis. Real-time quantitative PCR was performed by using the first-strand cDNA for RNA expression, using 250 nM forward and reverse primers, and the SYBR Green PCR master mix (BioRad). The reactions were performed by using the ABI 7900 Fast Real-Time PCR machine (Applied Biosystems). The primer sequences and PCR conditions are summarized in Supplementary Table 1. RNA expression levels were calculated as the relative expression ratio compared with human GAPDH.

### RACE PCR

GA007, CR3055, CR3056 RNA samples from PDX tumor were further examined by using the RACE (Rapid amplification of cDNA end) PCR kit SMARTer^®^RACE 5′/3′ Kit (Clontech). Procedure is as described by the manufacturer’s instructions. RACE PCR primers used include, RSPO2 GSP (Gene-Specific Primers):

1. 5′ RACE Rspo2 Exon 2 GATTACGCCAAGCTTGCGCTGCTGGGGAGGACTCAGAGGGAGAC, and
2. 5′ RACE Rspo2 Exon 3 GATTACGCCAAGCTTCGTCTCCATCGGTTGCCTTGGCAGTGGC.

### Nanostring Assay

Nanostring assay directly measures the gene expression by digital counting of nucleic acids, without RNA reverse transcription and PCR procedure. This assay employs nCounter Analysis System® which is based on a digital molecular barcoding technology, which uses molecular “barcodes” and microscopic imaging to detect and count up to several hundred unique transcripts in one hybridization reaction^40^. The assays were conducted by Wuxi AppTec (Shanghai) following the manufacturer’s manual.

### Cell lines, tumor samples and animal models

The Hek293 and LoVo cell lines were obtained from the ATCC. HEK293-TCF cell line was a gift from Curegenix (Guangzhou). Cells were cultured in relevant cell culture medium recommended by ATCC, supplemented with 10% (vol/vol) FBS.

All *in vivo* studies and establishments of various tumor models have been reviewed and approved by Institutional Animal Care and Use Committee (IACUC) of Shanghai Jiaotong University, under the IACUC protocol No. A2016039. All animal procedures and animal care were performed according to institutional animal research guidelines and were also in compliance with Shanghai Laboratory Animal Management Regulations. For setting up HEK293 cells-based xenograft models, the cells were expanded in culture, washed 3 times in PBS, then suspended at 5 × 10^7^ cells/ml density in the mixture of 50% 1640 culture medium and 50% Matrigel before inoculation in immune-deficient mice (SLAC Shanghai). When the tumor can be observed, their sizes were measured by caliper, calculated and expressed as tumor volume = (length × width^2^)/2^41,42^.

PDX mice were sacrificed to obtain the tumor tissues. Tumors were cut into pieces at about 3 mm^3^ in size and then re-implanted subcutaneously in 6–8 weeks nude mice (SLAC Shanghai) for *in vivo* studies. When the tumors reach to about 100–200 mm^3^ in size the compound efficacy studies were initiated.

### Western Blot

Antibodies were obtained from Abgent and GenSript: Anti-LGR5 (Abgent Cat#AM1992b, Abgent Cat# AP2745A), Anti-his (GenScript Cat#A00612). HEK293 and LoVo cells are dissociated in RAPID buffer addition of 1% PMSF (Beyotime). PDX samples tumor were homogenated in RAPID buffer. The protein extracts were combined with loading dye (Invitrogen), boiled and loaded onto 4–15% SDS PAGE gel (Invitrogen). After electrophoresis, proteins were transferred onto nitrocellulose membranes (Invitrogen) by using semidry Trans-Blot (Bio-Rad). The membrane was blocked by using 5% milk for 30 min, then incubated with the respective primary antibodies diluted in TBST (Tris-buffered saline Tween-20, containing 0.1% Tween-20 and 2% bovine serum albumin) overnight at 4 °C. The blots were washed and incubated with the appropriate secondary antibodies (Abcam) in TBST. Signals were detected by using the ECL plus Western Blotting Detection System (Tanon).

### Stable Transfected Cell Lines

HEK293-pcDNA-ER2, HEK293-pcDNA-R2 and HEK293-pcDNA3.1 cell lines were established by transfecting HEK293 cells with the respective plasmids using the Lipo2000 (Invitrogen) and selecting the stably transfected cells by G418 (ThermoFisher) selection at 800 ug/ml for 7 days, followed by incubations with G418 at the concentration of 200 ug/ml G418.

### Foci Formation Assay

The Foci formation experiments were done by seeding 3000 cells in each well of a 6-well-plate and cultured in 10% FBS 1% p/s 1640 culture medium with addition of 10 ng/ml RSPO2 (StemRD) in the experimental group until the colonies expand to 100–200 cells. The cells were fixed in 4% PFA, stained by crystal violet (Beyotime) and observed under a microscope. The Image J software was used to quantify the colonies number. T-test method is used for statistics.

### Cell Proliferation Assay

HEK293 or LoVo cells were seeded 10,000 cells into each well in the 96-well-plate with RSPO2 10 ng/ml conditioned medium. Cell proliferation was measured by CellTiter 96^®^ AQueous One Solution Cell Proliferation Assay kit (Promega), according to the manufacturer’s instructions.

### Luciferase Reporter Assay

HEK293-TCF reporter cells were transfected with pcDNA-ER2, pcDNA-R2 or pcDNA3.1 plasmids or treated with RSPO2 conditioned medium. Luciferase activity detection was done by microplate reader (Bio-Tek Instrument) and Luciferase Assay System kits (Promega), according to the manufacturer’s instructions.

### Migration Assays

The migration properties of the cells were assayed using the Transwell system. The 24-well Thanswell plates and chambers were from Foclon, with 900ul medium of 20% FBS + 1640 culture medium in the outer chamber. Thirty thousand cells were placed in 200ul DMEM medium without FBS, containing 0.5% BSA in the chamber. In the experiments group, 10 ng/ml RSPO2 was added in chambers, followed by culturing for 24 hrs in incubator 37 °C and 5% CO_2_. After 24 hrs, the chambers were taken out, washed in PBS twice times, and fixed by 4% PFA. Cells were stained by crystal violet for 10 min. Using cotton swab to remove the cell inside the chamber, it was turned over and migrated cell numbers were counted under microscope.

### CGX1321 Efficacy Studies *in vivo*

Compound CGX1321 is a gift from Curegenix (Guangzhou), Batch/Lot No.: PT-C14010931-D14001. The control formulation (D5W) for the vehicle group include 20% PEG400, 25% Solutol (20%) in 55% dextrose (5% in water) (v:v:v). There were prepared separately. The dose formulation was made as following: 1, Add appropriate amount of CGX1321 into an appropriate container; 2, Add 80% of final required volume of vehicle into the container; 3, Sonicate, and/or stir the mixture to form a homogeneous suspension; 4, Add remainder 20% of final required volume of vehicle to get the target concentration; 5, Sonicate the mixture to form a homogeneous suspension. In the efficacy studies, animals are dosed once a day (QD) via oral administration (p.o.). Tumor volumes were calculated based on the modified ellipsoidal formula, Endpoints for mice refer to the situation when tumor sizes reached above 2000 mm^3^ or when the studies were terminated.

### Statistical Analysis

Data are expressed as mean ± SED. Statistical analysis were performed using t-test or 2-way ANOVA using GraphPad prism 5 software (GraphPad Software).

## Electronic supplementary material

Supplementary Information

## References

[1] Clevers H (2006). Wnt/beta-catenin signaling in development and disease. Cell.

[2] Vogelstein B, Kinzler KW (2004). Cancer genes and the pathways they control. Nat Med.

[3] Monga S (2015). P. beta-Catenin Signaling and Roles in Liver Homeostasis, Injury, and Tumorigenesis. Gastroenterology.

[4] Fan K (2014). Wnt/beta-catenin signaling induces the transcription of cystathionine-gamma-lyase, a stimulator of tumor in colon cancer. Cell Signal.

[5] Shimokawa M (2017). Visualization and targeting of LGR5+ human colon cancer stem cells. Nature.

[6] Stewart DJ (2014). Wnt signaling pathway in non-small cell lung cancer. J Natl Cancer Inst.

[7] Uematsu K (2003). Activation of the Wnt pathway in non small cell lung cancer: evidence of dishevelled overexpression. Oncogene.

[8] Moon RT, Bowerman B, Boutros M, Perrimon N (2002). The promise and perils of Wnt signaling through beta-catenin. Science.

[9] Veeman MT, Axelrod JD, Moon RT (2003). A second canon: Functions and mechanisms of beta-catenin-independent wnt signaling. Dev Cell.

[10] Wodarz A, Nusse R (1998). Mechanisms of Wnt signaling in development. Annu Rev Cell Dev Bi.

[11] Cong F, Schweizer L, Varmus H (2004). Wnt signals across the plasma membrane to activate the beta-catenin pathway by forming oligomers containing its receptors, Frizzled and LRP. Development.

[12] Neth P (2006). Wnt signaling regulates the invasion capacity of human mesenchymal stem cells. Stem Cells.

[13] Mi K, Johnson GV (2005). Role of the intracellular domains of LRP5 and LRP6 in activating the Wnt canonical pathway. J Cell Biochem.

[14] Lemieux E, Cagnol S, Beaudry K, Carrier J, Rivard N (2015). Oncogenic KRAS signalling promotes the Wnt/beta-catenin pathway through LRP6 in colorectal cancer. Oncogene.

[15] MacDonald B. T., He X. (2012). Frizzled and LRP5/6 Receptors for Wnt/ -Catenin Signaling. Cold Spring Harbor Perspectives in Biology.

[16] Zorn AM (2001). Wnt signalling: Antagonistic Dickkopfs. Curr Biol.

[17] Kazanskaya O (2004). R-Spondin2 is a secreted activator of Wnt/beta-catenin signaling and is required for Xenopus myogenesis. Dev Cell.

[18] Schuijers J, Clevers H (2012). Adult mammalian stem cells: the role of Wnt, Lgr5 and R-spondins. EMBO J.

[19] Carmon KS, Gong X, Lin Q, Thomas A, Liu Q (2011). R-spondins function as ligands of the orphan receptors LGR4 and LGR5 to regulate Wnt/beta-catenin signaling. Proc Natl Acad Sci USA.

[20] Zebisch M (2013). Structural and molecular basis of ZNRF3/RNF43 transmembrane ubiquitin ligase inhibition by the Wnt agonist R-spondin. Nat Commun.

[21] Hao HX (2012). ZNRF3 promotes Wnt receptor turnover in an R-spondin-sensitive manner. Nature.

[22] de Lau W, Peng WC, Gros P, Clevers H (2014). The R-spondin/Lgr5/Rnf43 module: regulator of Wnt signal strength. Genes Dev.

[23] Seshagiri S (2012). Recurrent R-spondin fusions in colon cancer. Nature.

[24] Shinmura K (2014). RSPO fusion transcripts in colorectal cancer in Japanese population. Mol Biol Rep.

[25] Fischer MM (2017). RSPO3 antagonism inhibits growth and tumorigenicity in colorectal tumors harboring common Wnt pathway mutations. Sci Rep.

[26] Storm EE (2016). Targeting PTPRK-RSPO3 colon tumours promotes differentiation and loss of stem-cell function. Nature.

[27] Sekine S (2017). Comprehensive characterization of RSPO fusions in colorectal traditional serrated adenomas. Histopathology.

[28] Clevers H, Nusse R (2012). Wnt/beta-catenin signaling and disease. Cell.

[29] Kahn M (2014). Can we safely target the WNT pathway?. Nat Rev Drug Discov.

[30] Gurney A (2012). Wnt pathway inhibition via the targeting of Frizzled receptors results in decreased growth and tumorigenicity of human tumors. Proc Natl Acad Sci USA.

[31] Liu J (2013). Targeting Wnt-driven cancer through the inhibition of Porcupine by LGK974. Proc Natl Acad Sci USA.

[32] Anastas JN, Moon RT (2013). WNT signalling pathways as therapeutic targets in cancer. Nat Rev Cancer.

[33] Chen B (2009). Small molecule-mediated disruption of Wnt-dependent signaling in tissue regeneration and cancer. Nat Chem Biol.

[34] Duraiswamy AJ (2015). Discovery and Optimization of a Porcupine Inhibitor. Journal of medicinal chemistry.

[35] Jiang X (2013). Inactivating mutations of RNF43 confer Wnt dependency in pancreatic ductal adenocarcinoma. Proc Natl Acad Sci USA.

[36] C., K. S., Xing, G., Qiushi, L., Anthony, T. & Qingyun, L. R-spondins function as ligands of the orphan receptors LGR4 and LGR5 to regulate Wnt/β-catenin signaling (2011). 10.1073/pnas.1106083108 PMC3136304 21693646

[37] Menendez M (2004). Colorectal cancer risk and the APC D1822V variant. Int J Cancer.

[38] Feng M (2014). Association between the APC gene D1822V variant and the genetic susceptibility of colorectal cancer. Oncol Lett.

[39] Onuma K., Ochiai M., Orihashi K., Takahashi M., Imai T., Nakagama H., Hippo Y. (2013). Genetic reconstitution of tumorigenesis in primary intestinal cells. Proceedings of the National Academy of Sciences.

[40] Geiss GK (2008). Direct multiplexed measurement of gene expression with color-coded probe pairs. Nat Biotechnol.

[41] Euhus DM, Hudd C, LaRegina MC, Johnson FE (1986). Tumor measurement in the nude mouse. J Surg Oncol.

[42] Tomayko MM, Reynolds CP (1989). Determination of subcutaneous tumor size in athymic (nude) mice. Cancer Chemother Pharmacol.

