# Supplementary material for: Identification of RSPO2 Fusion Mutations and Target Therapy Using a Porcupine Inhibitor
Source: Sci Rep. 2018 Sep 24;8:14244. doi: 10.1038/s41598-018-32652-3 (PMC6155119; doi:10.1038/s41598-018-32652-3)
Supplement: Supplementary file 1 — Supplementary Information [file 41598_2018_32652_MOESM1_ESM.pdf]

Identification of RSPO2 Fusion Mutations and Target Therapy Using a Porcupine Inhibitor

Chong Li[1], Jing Cao[1], Ning Zhang[1], Shuang Wei[1], Xiaojing Chen[1] Meiqing Tu[1], Fengwei Xu and Yuhong Xu[1*]

[1] Pharmacy School, Shanghai Jiaotong University, Shanghai 200240, China
* Corresponding author. Email: yhxu@sjtu.edu.cn

S1

LGR5-F	CTGAACTAAGAACACTGA
LGR5-R	TTGAGGAAGAGATGAGAT
GAPDH-F	GAAGGTGAAGGTCGGAGT
GAPDH-R	GAAGATGGTGATGGGATTTC
RSPO2-F	ATCCGGGTACTATGGACACC
RSPO2-R	GCCTACTTTGCACTTGGTACA
Emc2-RSPO2 F	GGTTCTGGGAAGATGGCGAA
Emc2-RSPO2 R	ACTAGCTCGCTTACTGCGTC
β-catenin-F	GGTTGCCTTGCTCAACAAAA
β-catenin-R	TCCCAAGGAGACCTTCCATC

S2

Emc2 exon1-RSPO2 exon2

TCTCCACCGCCCGGTGGCGGGTCACGTGACTGCGTCTCCCCGCCCTCTCACCCCGCTGCCTCTAGGTTCTGGGAAGATGGCGAAGGTCTCAGAGC
TTTACGATGTCACTTGGGAAGGTTCGTGGCGGAGAGATGCTGATCGCGCTGAACTGACCGGTGCGGCCCGGGGGTGAGTGGCGAGTCTCCCTCT
GAGTCCTCCCCAGCAGCGCGGCCGGCGCCGGCTCTTTGGGCGAACCCTCCAGTTCCTAGACTTTGAGAGGCGTCTCTCCCCCGCCCGACCGCCCA
G**ATG**CAGTTTCGCCTTTTCTCCTTTGCCCTCATCATTCTGAACTGCATGGATTACAGCCACTGCCAAGGCAACCGATGGAGACGCAGTAAGCGAG
CTAGTTATGTATCAAATCCCATTTGCAAGGGTTGTTTGTCTTGTTCAAAG

HNF4G exon3-RSPO2 exon2

GAGCACCAGCGAAAGCAGCCAGTCTGAGATATTGACACTACAGAAAAAACTGACAGCTTACTCCTTGTATTGATTCTACTCTTCTCTACAAAT
ATAGACTCCGTTCCCTACCACAGCCTTGTTCGTGGCGGAGAGATGCTGATCGCGCTGAACTGACCGGTGCGGCCCGGGGGTGAGTGGCGAGTCT
CCCTCTGAGTCCTCCCCAGCAGCGCGGCCGGCGCCGGCTCTTTGGGCGAACCCTCCAGTTCCTAGACTTTGAGAGGCGTCTCTCCCCCGCCCGACC
GCCCAG**ATG**CAGTTTCGCCTTTTCTCCTTTGCCCTCATCATTCTGAACTGCATGGATTACAGCCACTGCCAAGGCAACCGATGGAGACGCAGTAA
GCGAGCTAGTTATGTATCAAATCCCATTTGCAAGGGTTGTTTGTCTTGTTCAAA

PVT1exon1-RSPO2 exon2

CTCCGGGCAGAGCGCGTGTGGCGGCCGAGCACATGGGCCCGCGGGCCGGGCGGGCTCGGGGCGGCCGGGACGAGGAGGGGCGACGACGAGCTGC
GAGCAAAGATGTGCCCCGGGACCCCCGGCACCTTCCAGTGGATTTCCTTGCGGAAAGGATGTTGGCGGTCCCTGTGACCTGTGGAGACACGGCC
AGATCTGCCCTCCAGTTCGTGGCGGAGAGATGCTGATCGCGCTGAACTGACCGGTGCGGCCCGGGGGTGAGTGGCGAGTCTCCCTCTGAGTCCTC
CCCAGCAGCGCGGCCGGCGCCGGCTCTTTGGGCGAACCCTCCAGTTCCTAGACTTTGAGAGGCGTCTCTCCCCCGCCCGACCGCCCAG**ATG**CAGT
TTCGCCTTTTCTCCTTTGCCCTCATCATTCTGAACTGCATGGATTACAGCCACTGCCAAGGCAACCGATGGAGACGCAGTAAGCGAGCTAGTTAT
GTATCAAATCCCATTTGCAAGGGTTGTTTGTCTTGTTCAAAGGACAATGGGTGTAGCCGATGTCAACAGAAGTTGTTCTTCTTCCTTCGAAGAG
AAGGGATGCGCCAGTATGGAGAGTGCCTGCAAGCTTGGC

PVT1 exon1-RSPO2 exon3

CTCCGGGCAGAGCGCGTGTGGCGGCCGAGCACATGGGCCCGCGGGCCGGGCGGGCTCGGGGCGGCCGGGACGAGGAGGGGCGACGACGAGCTG
CGAGCAAAGATGTGCCCCGGGACCCCCGGCACCTTCCAGTGGATTTCCTTGCGGAAAGGATGTTGGCGGTCCCTGTGACCTGTGGAGACACGG
CCAGATCTGCCCTCCACTAGTT**ATG**TATCAAATCCCATTTGCAAGGGTTGTTTGTCTTGTTCAAAGGACAATGGGTGTAGCCGATGTCAACAG
AAGTTGTTCTTCTTCCTTCGAAGAGAAGGGATGCGCCAGTATGGAGAGTGCCTGCA

S3: RACE PCR Program:

5 cycles:
94°C 30 sec
72°C 3 min
5 cycles:
94°C 30 sec
70°C 30 sec
72°C 3 min
20 cycles:
94°C 30 sec
68°C 30 sec
72°C 3 min

S4

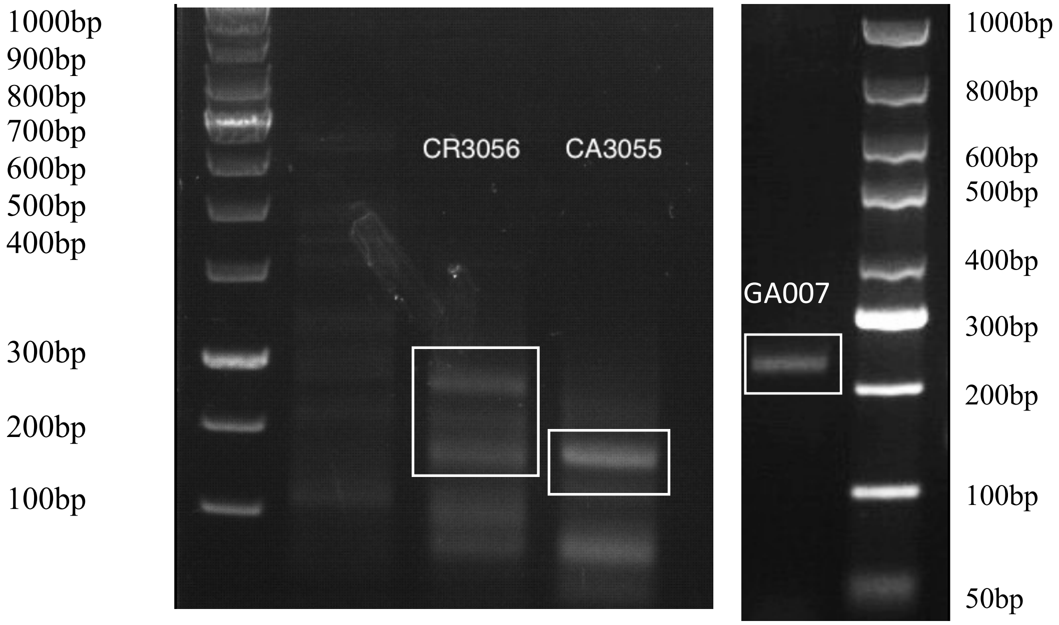

Supplementary information: S1, Primers were used in this paper. S2, RSPO2 fusions nucleic acid sequence. Yellow color is EMC2, HNF4G or PVT1 DNA sequence. Green color is RSPO2 DNA sequences. S3, RACE PCR program. S4, GA007, CR3056, GA3055 RACE PCR products full-length agarose gel electrophoresis.
